# Cameroonian professional soccer players and risk of atherosclerosis

**DOI:** 10.1186/s13104-017-2508-x

**Published:** 2017-06-02

**Authors:** Jobert Richie Nansseu, Vicky Jocelyne Ama Moor, Ruth Danielle M. Takam, Bertrand Zing-Awona, Marcel Azabji-Kenfack, Francine Tankeu, Corinne M. Tchoula, Bruno M. Moukette, Jeanne Y. Ngogang

**Affiliations:** 1 Department of Public Health, Faculty of Medicine and Biomedical Sciences, University of Yaoundé I, P. O. Box 1364, Yaoundé, Cameroon; 2 Department for the control of Diseases, Epidemics and Pandemics, Ministry of Public Health, Yaoundé, Cameroon; 3 Laboratory of Biochemistry, Yaoundé University Teaching Hospital, Yaoundé, Cameroon; 4 Department of Physiological Sciences and Biochemistry, Faculty of Medicine and Biomedical Sciences, University of Yaoundé I, Yaoundé, Cameroon; 5 Faculty of Sciences, University of Yaoundé I, Yaoundé, Cameroon; 6 University of Mountains, Bangangté, Cameroon; 7 Mathematical Engineering and Information System Laboratory, National Advanced School of Engineering, University of Yaoundé I, Yaoundé, Cameroon; 8 African Center of Excellence in Information and Communication Technologies, Yaoundé, Cameroon

**Keywords:** Atherosclerosis, Soccer players, Oxidized low-density lipoproteins, Antioxidants, Cameroon

## Abstract

**Background:**

Elevated titers of antibodies against oxidized low-density lipoproteins-cholesterol (ox-LDL-Ab) have been reported among professional athletes, paradoxically reflecting an increased risk of developing atherogenic and/or cardiovascular events. This study aimed to determine titers of ox-LDL-Ab in a group of Cameroonian professional soccer players, and evaluate their evolution during part of a competition season as well as the plasmatic antioxidant status to find out if this latter correlates with ox-LDL-Ab .

**Methods:**

We conducted a descriptive cohort study in 2012 including 18 healthy male soccer players. Three samplings were performed in March (T1), May (T2), and July 2012 (T3) to assess the lipid profile, titers of ox-LDL-Ab, and plasmatic concentrations of four antioxidants: the ferric reducing antioxidant power (FRAP), reduced glutathione (GSH), superoxide dismutase (SOD), and uric acid.

**Results:**

Ages ranged from 16 to 28 years with a median (interquartile range) of 19.5 (19–23) years. Total cholesterol, high-density lipoproteins-cholesterol (HDL-C), low-density lipoproteins-cholesterol (LDL-C) and triglycerides varied within normal ranges throughout the three samplings. While total cholesterol and LDL-C titers increased significantly (p = 0.003 and p = 0.006, respectively), triglycerides and HDL-C values varied non-significantly throughout the measurements (p = 0.061 and p = 0.192, respectively). The median ox-LDL-Ab titers were respectively: 653.3 (468.2–838.8) mIU/ml at T1, 777.7 (553.7–1150.7) mIU/ml at T2, and 1037.7 (901.7–1481.5) mIU/ml at T3. Overall, ox-LDL-Ab titers increased significantly from T1 to T3 (p = 0.006). Concomitantly, uric acid and FRAP concentrations decreased significantly (p = 0.001 and p = 0.003, respectively); on the contrary, GSH and SOD values increased, but insignificantly (p = 0.115 and p = 0.110, respectively). There was a positive and significant correlation between ox-LDL-Ab and HDL-C (ρ = 0.519, p = 0.027), and between ox-LDL-Ab and SOD (ρ = 0.504, p = 0.033) at T2. Ox-LDL-Ab values were expected to increase with each new visit (β = 201.1; p = 0.041) and each IU/ml of SOD titers (β = 23.6; p = 0.019).

**Conclusion:**

These Cameroonian professional soccer players exhibited high levels of ox-LDL-Ab reflecting elevated levels of oxidatively-modified LDL-C particles with an increment over time, this being insufficiently counterbalanced by the antioxidant defense mechanisms. As a consequence, they may be at increased atherogenic and cardiovascular risks.

## Background

Evidence has accumulated that oxidative stress may be implicated in the etiology of atherosclerosis. The risk of developing atherosclerosis is determined by the absolute levels of atherogenic lipoproteins, and by the relative tendency of such substances to undergo oxidation [1]. Specifically, low-density lipoproteins-cholesterol (LDL-C) are highly susceptible to oxidative processes initiated by oxygen free radicals, whereby oxidatively-modified LDL-C particles (ox-LDL) are produced as a result [2].

The arterial intima is the first site where oxidative modification of LDL-C occurs [2]. In fact, Ox-LDL particles constitute one of the major ligands for scavenger receptors on the arterial macrophage, which could account for foam cell formation [2]. Ox-LDL are strongly atherogenic and immunogenic [2, 3]. Consequently, autoantibodies against ox-LDL (ox-LDL-Ab) are produced by the immune system [2]. In vivo, elevated titers of ox-LDL-Ab have been found in many diseases such as atherosclerosis and coronary heart disease [3], hypertension [4], renal failure [5], and diabetes [6].

Physical activity is associated with beneficial changes in circulating lipids and lipoproteins [7, 8], body weight, blood pressure, insulin sensitivity [9], and coagulation parameters [10, 11]. Additionally, current data support that heavy endurance exercise increases the rate of oxygen consumption in humans up to 20-fold inducing reactive oxygen species (ROS) formation, and the removal of these formed species depends on antioxidant systems [12]. However, if the rise in the level of ROS exceeds the antioxidant capacity to neutralize them, as observed during strenuous aerobic exercise [13–15], or if antioxidant defenses are severely hampered, then cell lipids, proteins, and even DNA material may suffer oxidative damage [16]. Therefore, there may be an apparent paradox between the benefits of heavy aerobic exercise on cardiovascular risk factors and the potentially deleterious consequences of free radicals generated during intense aerobic exercise.

In this regard, professional athletes may be exposed to atherogenic and cardiovascular risks as it has already been suggested [17, 18]. In the present study, we determined the titers of ox-LDL-Ab in a group of professional soccer athletes playing in Cameroon, a Sub-Saharan African (SSA) country, and evaluated their evolution during part of a competition season as well as the antioxidant status to find out if this latter correlates with the immune response to oxidative modification of LDL-C.

## Methods

### Participants and setting

The procedures used in the present study are extensively described elsewhere [19]. We conducted a prospective and descriptive cohort study from May to July 2012 among a team of the Cameroon Elite one Football Championship, namely “Renaissance of Ngoumou”. This is a soccer club founded in 2000 and based in Ngoumou, a town situated 60 km from Yaoundé, the capital city of Cameroon.

Participants were male soccer players belonging to the just-cited team, enrolled if they were in good health and irrespective of their age, body mass index, region of origin and current diet. Before commencing the Championship in early March 2012, these players went through 14 weeks of intensive physical preparation consisting of at least 2 h of daily trainings plus regular friendly matches. Our study period covered the first leg of the Elite One Championship, part of the return phase, and a truce during which the team was engaged in qualification matches counting for the Cameroon Male Soccer National Cup. During this period, players were weekly subjected to 10 h of workout plus a match. Before enrolling a player in the study, we obtained a written and signed informed consent.

### Blood sampling

Three samplings were performed during the study period, occurring at an 8-week constant interval: March (T1), May (T2), and July 2012 (T3). The players had to be free of any training for at least 24 h prior to the blood collection, this being effectuated early in the morning after a 12-h overnight fasting. Blood was aseptically collected from each participant by venipuncture of the brachial vein in a 5 ml EDTA tube and in a 5 ml dry tube, without a tourniquet or fist clenching. Subsequently, samples were put on ice and immediately transported to the biochemistry laboratory where plasma and serum specimens were separated by centrifugation at 3000 rpm within 5 min and kept at −20 °C for further biochemical analyses not later than a week after sampling.

### Biochemical measurements

Each and every biochemical assay was performed at each sampling. Players’ lipid profile was measured in the serum using a spectrophotometer (Spectrophotometer 722–2000, MAIKONG Industry Co., Ltd., Shenzhen, China). Standard colorimetric methods served for the measurement of total cholesterol, triglycerides (TG) and high-density lipoproteins-cholesterol (HDL-C), with reagents from CYPRESS Diagnostics (CYPRESS Diagnostics, Langdorp, Belgium). LDL-C was derived based on the Friedwald formula.

Titers of ox-LDL-Ab were determined using commercial enzyme linked immunosurbant assay (ELISA) kits from Cusabio Biotech^®^ (Cusabio Biotech Company, Hubei, China), and were expressed in mIU/ml. A microplate reader (Reader IRE 96, SFRI Medical Diagnostics, Saint Jean d’Illac, France) served for the purpose. Normal values of Ox-LDL-Ab ranged between 200 and 600 mIU/ml in accordance with Pincemail et al. [17]. In order to evaluate the players’ anti-oxidant defense mechanisms, we measured the activity of superoxide dismutase (SOD) as well as the levels of uric acid, reduced glutathione (GSH) and that of the ferric reducing anti-oxidant power (FRAP). The procedures used for these assays were fully presented in a previous report [19]. The reading was performed on a spectrophotometer (Spectrophotometer 722–2000, MAIKONG Industry Co., Ltd., Shenzhen, China).

### Statistical methods

Statistical analyses were conducted using SPSS software (version 20.0, IBM SPSS^®^ Statistics, Chicago, Illinois, USA) and the R statistical package version 3.2.2 (The R Foundation for Statistical Computing, Vienna, Austria). Results are presented as count (proportion) and median (inter quartile range, IQR) where appropriate. The normality was tested, which was not effective; consequently, non-parametric tests were used. The Wilcoxon test served for the 2-paired sample comparisons, and the Friedman test was used to perform the overall trends analysis. The Spearman correlation test was utilized to seek for any association between quantitative variables.

The regression analysis was run using the mixed linear regression model. The initial model was as follows:

$$\begin{aligned} {\text{Ox-LDL-Ab}} &= {\text{b}}_{0} + {\text{b}}_{ 1} *\;{\text{visit}} + {\text{b}}_{ 2} *\;{\text{uric-acid}} + {\text{b}}_{ 3} *\;{\text{HDL}} + {\text{b}}_{ 4} *\;{\text{LDL}} \\ &\quad + {\text{b}}_{ 5} *\;{\text{triglycerides}} + \,{\text{b}}_{ 6} *\;{\text{FRAP}} +\, {\text{b}}_{ 7} *\;{\text{GSH}} \\ &\quad + {\text{b}}_{ 8} *\;{\text{SOD}} + {\text{b}}_{ 9} *\;{\text{age}} + {\text{b}}_{ 10} *\;\,{\text{BMI}} \\ &\quad + {\text{b}}_{ 1 1} *\;{\text{total cholesterol}} + {\text{b}}_{0 1} + {\text{b}}_{0 2} *\;{\text{visit}} \hfill \\ \end{aligned},$$

where b_1_–b_11_ were the coefficients of the fixed effects of the explanatory variables, and b_01_ and b_02_ were the coefficients of the random effects, respectively the random intercept and the random slope. Estimation of the model parameters was done using the restricted maximum likelihood method. Based on the analysis of variance (ANOVA) test, we ended up with the model containing the random slope and intercept and with the smallest akaike information criterion (AIC) when taking into account the correlation structure of random effects [Autoregressive (1)], ANOVA being insignificant when performing a 2-by-2 comparison of the models. Results were considered statistically significant each time the *p* value was less than 0.05.

### Ethical considerations

This study was granted an ethical clearance before initiation, delivered by the Cameroon National Ethics Committee for Human Health Research (No. 081/CNE/SE/2012). Additionally, we received approvals from the administrative staff of the team and from the Cameroon Football Federation. All procedures used in this survey were in keeping with the current revision of the Helsinki Declaration. On the other hand, all aspects and procedures of the study were fully presented and explained to each potential participant; we included only those who volunteered to take part in the study, who signed an informed consent accordingly. They were free to abandon the study at any moment without any prejudice.

## Results

Of the 30 players present at the first sampling, only 18 players attended the last measurement, hence a 40% proportion of abandon. Ages of participants ranged from 16 to 28 years with a median of 19.5 (19–23) years. The BMI varied between 21.4 and 25.8 kg/m^2^ with a median equal to 23.3 (22.4–24.4) kg/m^2^ (Table 1). Two subjects (11.1%) were slightly overweight (25 < BMI < 26 kg/m^2^).

**Table 1:** Overall results of the study participants at each measurement (N = 18)

Variable	Min	Max	Mean	SD	Median	IQR
Age (years)	16	28	20.6	3.1	19.5	19–23
BMI (kg/m^2^)	21.4	25.8	23.4	1.3	23.3	22.4–24.4
Total cholesterol 1 (mg/dl)	87	186	135	22	136	123–145
Total cholesterol 2 (mg/dl)	125	193	156	18	155	142–169
Total cholesterol 3 (mg/dl)	118	203	161	23	161	145–177
Triglycerides 1 (mg/dl)	61	138	84	21	76	69–99
Triglycerides 2 (mg/dl)	59	114	81	17	79	69–91
Triglycerides 3 (mg/dl)	68	127	96	19	93	83–116
HDL-C 1 (mg/dl)	35	85	63	13	64	51–72
HDL-C 2 (mg/dl)	53	90	66	11	65	55–72
HDL-C 3 (mg/dl)	40	81	58	11	58	49–63
LDL-C 1 (mg/dl)	20	101	56	20	55	41–69
LDL-C 2 (mg/dl)	38	104	74	19	79	56–87
LDL-C 3 (mg/dl)	50	134	82	21	81	70–96
ox-LDL-Ab1 (mIU/ml)	379.52	1786.20	724.69	366.65	653.32	468.18–838.80
ox-LDL-Ab2 (mIU/ml)	197.52	1494.34	832.10	377.54	777.73	553.71–1150.74
ox-LDL-Ab3 (mIU/ml)	284.78	2640.76	1198.67	564.05	1037.68	901.74–1481.46
Uric acid 1 (mg/l)	32.45	115.47	61.67	20.05	58.11	49.05–70.00
Uric acid 2 (mg/l)	26.46	63.38	48.44	9.58	48.51	41.98–54.27
Uric acid 3 (mg/l)	42.84	86.81	57.54	10.87	56.74	48.39–63.97
FRAP 1 (mmol/l)	0.405	0.658	0.539	0.082	0.537	0.469–0.613
FRAP 2 (mmol/l)	0.285	0.597	0.441	0.084	0.451	0.397–0.488
FRAP 3 (mmol/l)	0.204	0.616	0.470	0.126	0.503	0.363–0.575
GSH 1 (µmol/l)	1.20	7.35	3.71	1.61	3.08	2.61–4.83
GSH 2 (µmol/l)	1.39	6.61	3.38	1.40	3.05	2.44–4.39
GSH 3 (µmol/l)	1.98	7.94	3.84	1.42	3.64	2.72–4.46
SOD 1 (IU/ml)	4.95	26.45	15.35	6.70	17.25	8.27–20.99
SOD 2 (IU/ml)	5.63	26.45	14.23	6.62	14.38	7.60–20.11
SOD 3 (IU/ml)	4.44	29.90	18.05	8.57	21.25	6.88–24.44

*Min* minimum, *Max* maximum, *SD* standard deviation, *IQR* inter quartile range, *BMI* body mass index

The median levels of total cholesterol, HDL-C, LDL-C and TG were both normal throughout the three samplings (Table 1). Total cholesterol and LDL-C significantly increased from T1 to T3: median 136 vs. 161 mg/dl; p = 0.003 and 55 vs. 81 mg/dl; p = 0.006, respectively. Contrariwise, TG and HDL-C titers did not vary significantly throughout the 3 measurements: p = 0.061 and p = 0.192 respectively (Tables 1, 2).

**Table 2:** Comparison of variables between the 3 measurements (T1, T2 and T3)

Comparison	p value^a^	Overall comparison (p value)^b^
Total cholesterol 1–total cholesterol 2	0.006*	
Total cholesterol 2–total cholesterol 3	0.257	0.003*
Total cholesterol 1–total cholesterol 3	0.004*	
Triglycerides 1–triglycerides 2	0.794	
Triglycerides 2–triglycerides 3	0.018*	0.061
Triglycerides 1–triglycerides 3	0.094	
HDL-C 1–HDL-C 2	0.381	
HDL-C 2–HDL-C 3	0.089	0.192
HDL-C 1–HDL-C 3	0.107	
LDL-C 1–LDL-C 2	0.008*	
LDL-C 2–LDL-C 3	0.064	0.006*
LDL-C 1–LDL-C 3	0.002*	
ox-LDL-Ab 1–ox-LDL-Ab 2	0.122	
ox-LDL-Ab 2–ox-LDL-Ab 3	0.010*	0.006*
ox-LDL-Ab 1–ox-LDL-Ab 3	0.006*	
Uric acid 1–uric acid 2	0.001*	
Uric acid 2–uric acid 3	0.002*	0.001*
Uric acid 1–uric acid 3	0.557	
FRAP 1–FRAP 2	0.004*	
FRAP 2–FRAP 3	0.528	0.003*
FRAP 1–FRAP 3	0.035*	
GSH 1–GSH 2	0.663	
GSH 2–GSH 3	0.206	0.115
GSH 1–GSH 3	0.306	
SOD 1–SOD 2	0.170	
SOD 2–SOD 3	0.007*	0.110
SOD 1–SOD 3	0.065	

* p value <0.05

^a^These 2-paired comparisons were performed using the Wilcoxon test

^b^These are the results of overall comparisons of the 3 measurements using the Friedman test

**Fig. 1:**
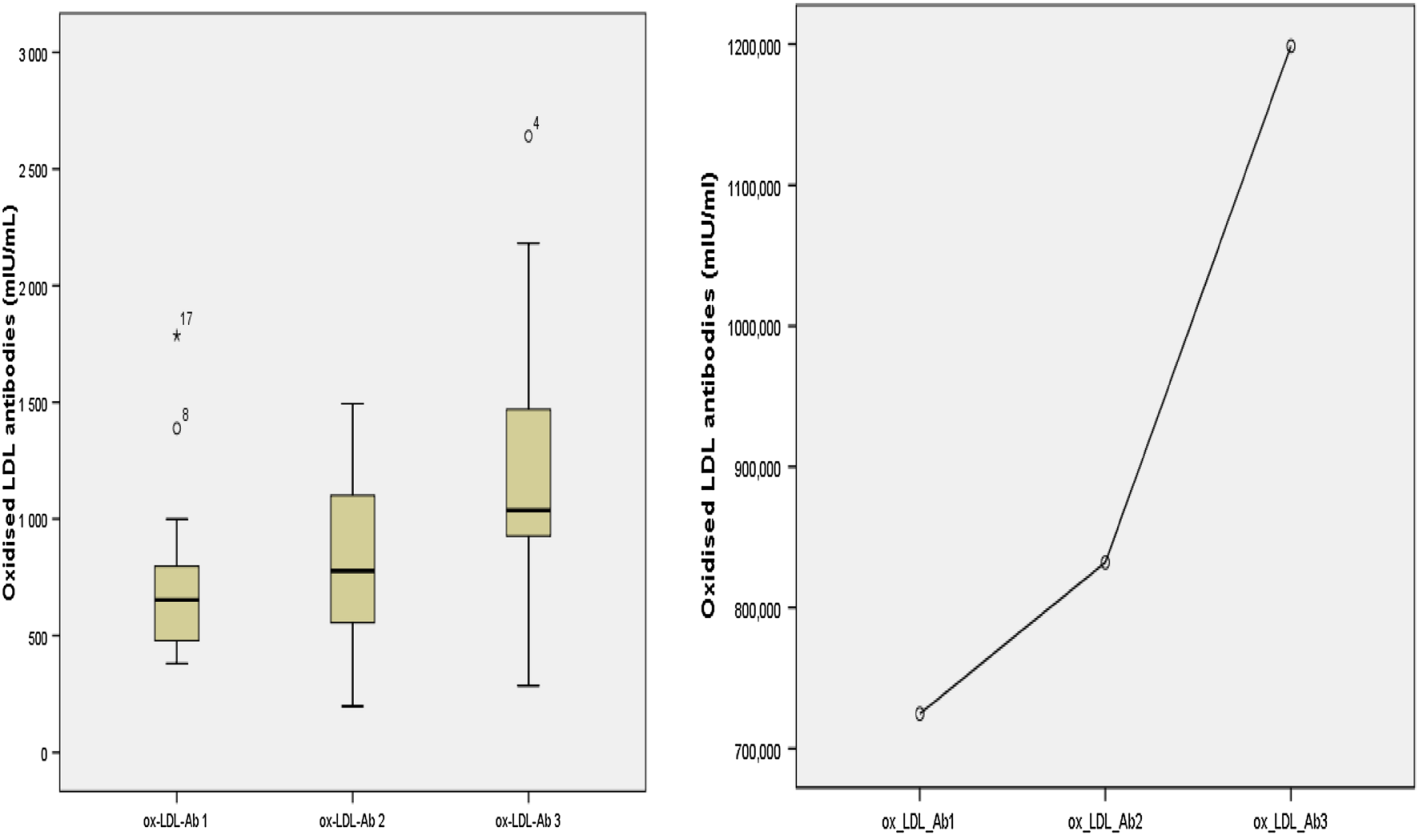
Variation of oxidized Low-Density-Lipoprotein antibodies (ox-LDL-Ab) titer between the three measurements. From T1 to T3, we observed a significant rise in oxidized LDL antibodies titers (p = 0.006)

The median ox-LDL-Ab titers were high during the three samplings: 653.3 (468.2–838.8) mIU/ml at T1, 777.7 (553.7–1150.7) mIU/ml at T2, and 1037.7 (901.7–1481.5) mIU/ml at T3 (Table 1). Around 56% of participants had a titer greater than 600 mIU/ml at T1, 72.2% at T2, and 88.9% at T3, with very high titers approaching 3000 mIU/ml. Ox-LDL-Ab values raised insignificantly from T1 to T2 (p = 0.122), but the increment was significant between T2 and T3 (p = 0.010) and between T1 and T3 (p = 0.006). The overall trend throughout the three measurements was a statistically significant increment: p = 0.006 (Tables 1, 2; Fig. 1). Concomitantly, uric acid concentrations decreased significantly throughout the 3 samplings (median: 58.11 vs. 56.74 mg/l; p = 0.001), as well as FRAP titers (median 0.54 vs. 0.50 mmol/l; p = 0.003). On the contrary, GSH and SOD values increased (median 3.08 vs. 3.64 µmol/l and 17.25 vs. 21.25 IU/ml respectively), but non-significantly: p = 0.115 and p = 0.110 respectively (Tables 1, 2).

There was a positive and significant correlation between ox-LDL-Ab and HDL-C, but only at the second sampling (ρ = 0.519, p = 0.027). There was also a positive and significant correlation between ox-LDL-Ab and SOD at the second sampling (ρ = 0.504, p = 0.033). The other correlations between ox-LDL-Ab and antioxidants were found very weak and statistically non-significant (Table 3).

**Table 3:** Correlations between oxidized LDL antibodies (ox-LDL-Ab) and the other parameters at the three samplings (T1, T2 and T3)

Sampling	Correlation between ox-LDL-Ab and	Coefficient of correlation (ρ)	p value
T1	Total cholesterol	0.316	0.201
	Triglycerides	−0.138	0.586
	HDL	0.026	0.919
	LDL	0.368	0.133
	Uric acid	0.047	0.855
	FRAP	−0.413	0.089
	GSH	0.241	0.336
	SOD	0.136	0.591
T2	Total cholesterol	0.197	0.434
	Triglycerides	−0.115	0.650
	HDL	*0.519*	*0.027* *
	LDL	−0.146	0.564
	Uric acid	−0.094	0.711
	FRAP	0.277	0.266
	GSH	0.164	0.515
	SOD	*0.504*	*0.033* *
T3	Total cholesterol	−0.218	0.385
	Triglycerides	0.003	0.990
	HDL	0.085	0.738
	LDL	−0.250	0.317
	Uric acid	−0.123	0.627
	FRAP	−0.212	0.399
	GSH	0.100	0.692
	SOD	0.085	0.738

* p value <0.05

Table 4 depicts the results of the regression analysis. All the coefficients of correlation were less than 0.566, reflecting an absence of strong correlation between the explanatory variables in the model. Two variables were significantly associated with ox-LDL-Ab: visit and SOD. The significance of the variable “visit” reflects a time effect: at each new visit, it is expected on average a 201.1 mIU/ml increase in ox-LDL-Ab levels (p = 0.041). Likewise, ox-LDL-Ab titers are expected to rise by 23.6 mIU/ml with each SOD increment of 1 IU/ml (p = 0.019). The coefficient of correlation between random slope and intercept was −0.92, and the AIC of this final model was 701.66.

**Table 4:** Results of the mixed linear regression model analysis with oxidized low-density lipoproteins (ox-LDL-Ab) as the dependent variable

Variable	Value (β)	Standard error	p value
Visit	*201.09*	*93.86*	0.0413*
Uric acid	7.53	4.47	0.1037
HDL	714.81	478.78	0.1470
LDL	377.35	312.03	0.2370
Triglycerides	−38.55	322.16	0.9056
FRAP	129.60	608.49	0.8329
GSH	59.49	40.86	0.1569
SOD	*23.63*	*9.47*	0.0191*
Age	26.41	21.86	0.2445

* p < 0.05

## Discussion

Results from the present study show that titers of ox-LDL-Ab are very high in most of our players, with a significant increment over time as the competition is evolving. It seems therefore justified to infer that these high titers could be accounted for by a higher in vivo susceptibility of LDL-C to structural modification under conditions of intensive training-induced oxidative stress. Furthermore, of the 4 antioxidants measured, only SOD values could impact that of ox-LDL-Ab, and there were no or very weak correlations between ox-LDL-Ab and these antioxidants throughout the samplings. Consequently, the over-production of ox-LDL, inferred from that of ox-LDL-Ab, may not be efficiently counterbalanced by the antioxidant defense mechanism, which may lead thereby to lipid peroxidation and cell damage.

High levels of TG and low levels of HDL-C are among the elements shaping atherogenic dyslipidemia (AD); along with elevated levels of LDL-C, they are recognized as independent risk factors for cardiovascular disease (CVD) [20]. There is body of evidence bolstering that regular physical training is associated with lowering of plasma total TG, total cholesterol and LDL-C concentrations, and an increase in HDL-C titers [7], hence the prevention or retardation of AD, and CVD as well. We found for instance low levels of total cholesterol, LDL-C, and TG, and high levels of HDL-C among our players and throughout the different samplings, concurring previous reports [7, 21]. However, we observed a significant increment in levels of total cholesterol and LDL-C, a non-significant increase in TG concentrations, and an insignificant diminution in HDL-C titers over time, though their median values remained under normal ranges. As we did not compare our players to healthy sedentary individuals, and did not assess their nutritional profile, it is therefore difficult to have a clear explanation of this finding. The need to undertake a well-designed study in order to thoroughly assess this issue is thereby warranted.

Concurring with previous reports [2, 14, 17, 18, 22], we observed high titers of ox-LDL-Ab as well as an increment in these titers over time. For instance, Pincemail et al. [17] found very high titers of ox-LDL-Ab among half of their athletes, and Shippinger et al. [18] observed an increase in ox-LDL-Ab at the mid-competition time point, though the values levelled off thereafter. On the contrary, other studies revealed a significant reduction in the oxidation of LDL-C after physical activity [21, 23], but these studies were undertaken in non-athletes. Our findings do suggest the presence of in vivo LDL-C oxidation processes during chronic exhaustive exercise.

Ox-LDL particles have atherogenic properties, and elevated levels of circulating ox-LDL have been reported to predict future cardiovascular events [23, 24]. Indeed, a large number of epidemiological and clinical studies indicate that increased titers of ox-LDL-Ab correlated with the progression and intensity of atherosclerosis [25], hypertension [4], and the appearance of coronary artery diseases [26]. Ox-LDL-Ab have been found positively correlated with the degree of carotid stenosis in patients undergoing endarterectomy [27], and the ox-LDL/LDL-C ratio was significantly associated with the coronary artery calcification score in hemodialysis patients [28]. There is therefore an apparent paradox between the benefits of heavy aerobic exercise on cardiovascular risk factors and the potentially deleterious consequences of free radicals generated during heavy exercise. According to such observation, we can hypothesize that atherosclerotic diseases in professional athletes cannot be excluded during or at the end of their careers as it has been shown that atherogenic and cardiovascular risks are plausible in these categories of top athletes.

While examining the antioxidant response to the production of pro-oxidants (herein ox-LDL-Ab), we intriguingly noticed that apart from SOD levels which increased over time and impacted ox-LDL-Ab variation (β = 23.6; p = 0.019), GSH, FRAP and uric acid values decreased. Furthermore, there were no or very weak correlations between ox-LDL-Ab and other antioxidants at the different samplings. These findings suggest that the production of pro-oxidants among our soccer players may not be efficiently counterbalanced by the anti-oxidant defense mechanisms. Mirroring our results, Lehki et al. [13] observed that alterations in the activities of SOD and higher level of non-enzymatic defenses in trained subjects may not be sufficient enough to counteract the increase in ROS produced by endurance training. By contrast, Brites et al. [29] and Liu et al. [14] showed that the increased production of pro-oxidants was accompanied by an adaptive response of antioxidants. These discrepancies may be explained by the different designs of these studies, the intensity of physical activity, and differences in diets.

Based on our results showing high levels of circulating ox-LDL-Ab insufficiently counteracted by antioxidant production among our athletes, it is likely that they may be exposed to lipid peroxidation and cell damage, hence an increased risk of atherosclerosis and CVD as well. In this context, supplementation of antioxidants could be beneficial in this setting. Indeed, appropriate nutrition is likely to be vitally important in maintaining adequate antioxidant defense mechanisms [30]. There is convincing evidence that vitamin C & E supplementation have decreased LDL-C susceptibility to oxidation after exercise, though they did not enhance performance [15, 30–32]. Furthermore, Ibero-Baraibar et al. [33] demonstrated that the consumption of cocoa extract as part of ready-to-eat meals and within a hypo-caloric diet significantly decreased ox-LDL levels in middle-aged subjects, these effects being more beneficial in men. Contrariwise, some authors have found that, except for carbohydrate beverages, none of the commonly-used supplements are an effective countermeasure to exercise-induced immune suppression [34, 35]. Additionally, strong measures should be put in place to promote the adoption of healthy diets among our players. Moreover, further studies are needed, especially in SSA athletes, to better assess the cost-benefit profile of supplementation with exogenous antioxidants.

Unfortunately, the absence of a control group composed of healthy sedentary subjects precluded us from comparing and contrasting results that would have been obtained from the two groups. As a consequence, we are unable to conclude with strong evidence that changes observed with regard to pro- and antioxidant substances measured over time are only accounted for by chronic exposure to strenuous exercise. Further well-designed studies with more subjects are warranted to better elucidate the effect of chronic exhaustive training on oxidative stress status of professional SSA athletes. Additionally, we did not investigate the nutritional profile of our participants which could have explained some of our results, notably the variations in uric acid levels, even though the players’ kidney function was normal [36]. Another flaw of this study lies in the small number of participants rending difficult the application of the mixed linear regression model. Nonetheless, we applied the restricted maximum likelihood method which is well suited for small samples. On the other hand and in order to better assess the antioxidant defense mechanism of our participants, we measured 4 related markers in line with the recommendations stating that at least two techniques should be used for an accurate and consistent evaluation of oxidative stress in humans [37]. Moreover, there are few studies that have followed-up professional athletes during part of a competition to seek for modifications of their oxidative status over time, and to the best of our knowledge, this is one of the rare studies conducted in SSA and dedicated to this question.

## Conclusion

This study showed that in a context of chronic strenuous exercise, Cameroonian professional soccer players exhibit high levels of ox-LDL-Ab with an increment in these titers over time, this being insufficiently counterbalanced by their antioxidant defense mechanisms. As a consequence, atherogenic and cardiovascular risks are plausible in these categories of top athletes. Therefore, indices of oxidative stress as well as the antioxidant capacity should be monitored in this population. Additionally, bolstering athletes’ antioxidant defenses with exogenous antioxidant supplements may ameliorate exercise-induced damage (LDL-C oxidation), hence avoiding lipid peroxidation and tissue damage, but this must be underpinned by local robust evidence. Further studies are warranted, which will assess the global risk of atherosclerosis among our top athletes, including all its known risk factors.

## Abbreviations

FRAP: ferric reducing antioxidant power of plasma
GSH: reduced glutathione
HDL-C: high-density lipoproteins-cholesterol
LDL-C: low-density lipoproteins-cholesterol
Ox-LDL: oxidized low-density lipoproteins-cholesterol
Ox-LDL-Ab: oxidized low-density lipoproteins antibodies
ROS: reactive oxygen species
SOD: superoxide dismutase
SSA: sub-Saharan Africa
TG: triglycerides

## References

[1] Schreier L, Sanguinetti S, Mosso H, Lopez G, Siri L, Wikinski RLW (1996). Low-density lipoprotein composition and oxidability in atherosclerotic cardiovascular disease. Clin Biochem.

[2] Kłapcińska B, Kempa K, Sobczak A, Sadowska-Krepa E, Jagsz S, Szołtysek I (2005). Evaluation of autoantibodies against oxidized LDL (oLAB) and blood antioxidant status in professional soccer players. Int J Sports Med.

[3] Inoue T, Uchida T, Kamishirado H, Takayanagi K, Hayashi T, Morooka S (2001). Clinical significance of antibody against oxidized low density lipoprotein in patients with atherosclerotic coronary artery disease. J Am Coll Cardiol.

[4] Maggi E, Marchesi E, Ravetta V, Martignoni A, Finardi G, Bellomo G (1995). Presence of autoantibodies against oxidatively modified low-density lipoprotein in essential hypertension: a biochemical signature of an enhanced in vivo low-density lipoprotein oxidation. J Hypertens.

[5] Shoji T, Fukumoto M, Kimoto E, Shinohara K, Emoto M, Tahara H, Koyama H, Ishimura E, Nakatani T, Miki T (2002). Antibody to oxidized low-density lipoprotein and cardiovascular mortality in end-stage renal disease. Kidney Int.

[6] Mironova MA, Klein RL, Virella GT, Lopes-Virella MF (2000). Anti-modified LDL antibodies, LDL-containing immune complexes, and susceptibility of LDL to in vitro oxidation in patients with type 2 diabetes. Diabetes.

[7] Durstine JL, Haskell WL (1994). Effects of exercise training on plasma lipids and lipoproteins. Exerc Sport Sci Rev.

[8] Pronk NP (1993). Short term effects of exercise on plasma lipids and lipoproteins in humans. Sports Med.

[9] Hardin DS, Azzarelli B, Edwards J, Wigglesworth J, Maianu L, Brechtel G, Johnson A, Baron A, Garvey WT (1995). Mechanisms of enhanced insulin sensitivity in endurance-trained athletes: effects on blood flow and differential expression of GLUT 4 in skeletal muscles. J Clin Endocrinol Metab.

[10] Sayed MS (1996). Effects of exercise on blood coagulation, fibrinolysis and platelet aggregation. Sports Med.

[11] Ernst E, Resch KL (1995). Therapeutic interventions to lower plasma fibrinogen concentration. Eur Heart J.

[12] Powers SK, Ji LL, Leeuwenburgh C (1999). Exercise training-induced alterations in skeletalmuscle antioxidant capacity: a brief review. Med Sci Sports Exerc.

[13] Lekhi C, Gupta PH, Singh B (2007). Influence of exercise on oxidant stress products in elite Indian cyclists. Br J Sports Med.

[14] Liu ML, Bergholm R, Mäkimattila S, Lahdenperä S, Valkonen M, Hilden H, Yki-Järvinen H, Taskinen MR (1999). A marathon run increases the susceptibility of LDL to oxidation in vitro and modifies plasma antioxidants. Am J Physiol.

[15] Clarkson PM, Thompson HS (2000). Antioxidants: what role do they play in physical activity and health?. Am J Clin Nutr.

[16] Packer L (1997). Oxidants, antioxidant nutrients and the athlete. J Sports Sci.

[17] Pincemail J, Lecomte J, Castiau J, Collard E, Vasankari T, Cheramy-Bien J, Limet R, Defraigne J (2000). Evaluation of autoantibodies against oxidized LDL and antioxidant status in top soccer and basketball players after 4 months of competition. Free Radic Biol Med.

[18] Schippinger G, Wonisch W, Abuja PM, Fankhauser F, Winklhofer-Roob BM, Halwachs G (2002). Lipid peroxidation and antioxidant status in professional American football players during competition. Eur J Clin Invest.

[19] Takam RD, Ama Moor VJ, Nansseu JR, Pieme CA, Azabji-Kenfack M, Moukette BM, Tankeu F, Tcoula CM, Ngogang JY (2015). Effects of chronic strenuous physical exercise on oxidative stress and antioxidant capacity in sub-Saharan African professional soccer players. EJSM..

[20] Manjunath CN, Rawal JR, Irani PM, Madhu K (2013). Atherogenic dyslipidemia. Indian J Endocrinol Metab.

[21] Kujala UM, Ahotupa M, Vasankari T, Kaprio J, Tikkanen MJ (1996). Low LDL oxidation in veteran endurance athletes. Scand J Med Sci Sports.

[22] Sánchez-Quesada JL, Homs-Serradesanferm R, Serrat-Serrat J, Serra-Grima JR, González-Sastre F, Ordóñez-Llanos J (1995). Increase of LDL susceptibility to oxidation occurring after intense, long duration aerobic exercise. Atherosclerosis.

[23] Park JH, Miyashita M, Takahashi M, Harada K, Takaizumi K, Kim HS, Suzuki K, Nakamura Y (2011). Oxidised low-density lipoprotein concentrations and physical activity status in older adults: the WASEDA active life study. J Atheroscler Thromb.

[24] Pohjantähti-Maaroos H, Palomäki A (2011). Comparison of metabolic syndrome subjects with and without erectile dysfunction–levels of circulating oxidised LDL and arterial elasticity. Int J Clin Pract.

[25] Bui MN, Sack MN, Moutsatsos G, Lu DY, Katz P, McCown R, Breall JA, Rackley CE (1996). Autoantibody titers to oxidized low-density lipoprotein in patients with coronary atherosclerosis. Am Heart J.

[26] Lehtimäki T, Lehtinen S, Solakivi T, Nikkilä M, Jaakkola O, Jokela H, Ylä-Herttuala S, Luoma JS, Koivula T, Nikkari T (1999). Autoantibodies against oxidized low density lipoprotein in patients with angiographically verified coronary artery disease. Arterioscler Thromb Vasc Biol.

[27] Chiesa R, Melissano G, Castellano R, Astore D, Marone EM, Grossi A, Maggi E, Finardi G, Casasco A, Bellomo G (1998). In search of biological markers of high-risk carotid artery atherosclerotic plaque: enhanced LDL oxidation. Ann Vasc Surg.

[28] Asamiya Y, Yajima A, Tsuruta Y, Otsubo S, Nitta K (2013). Oxidised LDL/LDL-cholesterol ratio and coronary artery calcification in haemodialysis patients. Nutr Metab Cardiovasc Dis.

[29] Brites FD, Evelson PA, Christiansen MG, Nicol MF, Basílico MJ, Wikinski RW, Llesuy SF (1999). Soccer players under regular training show oxidative stress but an improved plasma antioxidant status. Clin Sci (Lond).

[30] Gravina L, Ruiz F, Diaz E, Lekue JA, Badiola A, Irazusta J, Gil SM (2012). Influence of nutrient intake on antioxidant capacity, muscle damage and white blood cell count in female soccer players. J Int Soc Sports Nutr.

[31] Zoppi CC, Hohl R, Silva FC, Lazarim FL, Neto JM, Stancanneli M, Macedo DV (2006). Vitamin C and e supplementation effects in professional soccer players under regular training. J Int Soc Sports Nutr.

[32] Sánchez-Quesada JL, Jorba O, Payés A, Otal C, Serra-Grima R, González-Sastre F, Ordóñez-Llanos J (1998). Ascorbic acid inhibits the increase in low-density lipoprotein (LDL) susceptibility to oxidation and the proportion of electronegative LDL induced by intense aerobic exercise. Coron Artery Dis.

[33] Ibero-Baraibar I, Abete I, Navas-Carretero S, Massis-Zaid A, Martinez JA, Zulet MA (2014). Oxidised LDL levels decreases after the consumption of ready-to-eat meals supplemented with cocoa extract within a hypocaloric diet. Nutr Metab Cardiovasc Dis.

[34] Nieman DC (2000). Exercise immunology: future directions for research related to athletes, nutrition, and the elderly. Int J Sports Med.

[35] Nieman DC (2001). Exercise immunology: nutritional countermeasures. Can J Appl Physiol.

[36] Ama Moor VJ, Tankeu F, Pieme CA, Takam Mafoche RD, Nansseu Njingang JR, Moukette Moukette B, Ngogang J. Biological Monitoring of a Group of Cameroonian Soccer Players. Health Sci Dis. 2014; 15: 2. http://www.hsd-fmsb.org. **(In French)**.

[37] Yu BP, Suescun EA, Yang SY (1992). Effect of age-related lipid peroxidation on membrane fluidity and phospholipase A2: modulation by dietary restriction. Mech Ageing Dev.

